# Validation of the isothermal *Schistosoma haematobium* Recombinase Polymerase Amplification (RPA) assay, coupled with simplified sample preparation, for diagnosing female genital schistosomiasis using cervicovaginal lavage and vaginal self-swab samples

**DOI:** 10.1371/journal.pntd.0010276

**Published:** 2022-03-14

**Authors:** John Archer, Farhan K. Patwary, Amy S. Sturt, Emily L. Webb, Comfort Rutty Phiri, Tobias Mweene, Richard J. Hayes, Helen Ayles, Eric A. T. Brienen, Lisette van Lieshout, Bonnie L. Webster, Amaya L. Bustinduy

**Affiliations:** 1 Wolfson Wellcome Biomedical Laboratories, Department of Zoology, Natural History Museum, Cromwell Road, London, United Kingdom; 2 Department of Parasitology, Liverpool School of Tropical Medicine, Liverpool, United Kingdom; 3 Department of Clinical Research, London School of Hygiene and Tropical Medicine, Keppel Street, London, United Kingdom; 4 Department of Infectious Diseases Epidemiology, London School of Hygiene and Tropical Medicine, Keppel Street, London, United Kingdom; 5 School of Medicine, University of Zambia, Zambart, Lusaka, Zambia; 6 Department of Parasitology, Leiden University Medical Center, Leiden, the Netherlands; Universidade Federal de Alagoas - Campus Arapiraca, BRAZIL

## Abstract

**Background:**

Female genital schistosomiasis (FGS) is a neglected and disabling gynecological disease that can result from infection with the parasitic trematode *Schistosoma haematobium*. Accurate diagnosis of FGS is crucial for effective case management, surveillance and control. However, current methods for diagnosis and morbidity assessment can be inaccessible to those at need, labour intensive, costly and unreliable. Molecular techniques such as PCR can be used to reliably diagnose FGS via the detection of *Schistosoma* DNA using cervicovaginal lavage (CVL) samples as well as lesser-invasive vaginal self-swab (VSS) and cervical self-swab samples. PCR is, however, currently unsuited for use in most endemic settings. As such, in this study, we assessed the use of a rapid and portable *S*. *haematobium* recombinase polymerase amplification (Sh-RPA) isothermal molecular diagnostic assay, coupled with simplified sample preparation methodologies, to detect *S*. *haematobium* DNA using CVL and VSS samples provided by patients in Zambia.

**Methodology/Principal findings:**

VSS and CVL samples were screened for FGS using a previously developed Sh-RPA assay. DNA was isolated from VSS and CVL samples using the *QIAamp Mini kit* (n = 603 and 527, respectively). DNA was also isolated from CVL samples using two rapid and portable DNA extraction methods: 1) the *SpeedXtract Nucleic Acid Kit* (n = 223) and 2) the *Extracta DNA Tissue Prep Kit* (n = 136). Diagnostic performance of the Sh-RPA using VSS DNA extacts (*QIAamp Mini kit*) as well as CVL DNA extracts (*QIAamp Mini kit*, *SpeedXtract Nucleic Acid Kit* and *Extracta DNA Tissue Prep Kit*) was then compared to a real-time PCR reference test.

Results suggest that optimal performance may be achieved when the Sh-RPA is used with PuVSS samples (sensitivity 93.3%; specificity 96.6%), however no comparisons between different DNA extraction methods using VSS samples could be carried out within this study. When using CVL samples, sensitivity of the Sh-RPA ranged between 71.4 and 85.7 across all three DNA extraction methods when compared to real-time PCR using CVL samples prepared using the *QIAamp Mini kit*. Interestingly, of these three DNA extraction methods, the rapid and portable *SpeedXtract* method had the greatest sensitivity and specificity (85.7% and 98.1%, respectively). Specificity of the Sh-RPA was >91% across all comparisons.

**Conclusions/Significance:**

These results supplement previous findings, highlighting that the use of genital self-swab sampling for diagnosing FGS should be explored further whilst also demonstrating that rapid and portable DNA isolation methods can be used to detect *S*. *haematobium* DNA within clinical samples using RPA. Although further development and assessment is needed, it was concluded that the Sh-RPA, coupled with simplified sample preparation, shows excellent promise as a rapid and sensitive diagnostic tool capable of diagnosing FGS at the point-of-care in resource-poor schistosomiasis-endemic settings.

## Author summary

Female genital schistosomiasis (FGS) is a neglected gynecological disease that can result from infection with the parasitic trematode *Schistosoma haematobium*. In addition to disabling FGS-associated pathologies, *S*. *haematobium* infection has also been associated with sexually transmitted infections (STIs), cervical dysplasia and HIV transmission.Accurate diagnosis of FGS is crucial for effective case management, surveillance and control. However, current methods for diagnosis and morbidity assessment can be inaccessible to those at need, labour intensive, costly and unreliable. Molecular techniques such as PCR can be used to reliably diagnose FGS via the detection of *Schistosoma* DNA using cervicovaginal lavage (CVL) samples as well as lesser-invasive vaginal self-swab (VSS) and cervical self-swab samples. PCR is, however, currently unsuited for use in most endemic settings. As such, in this study, we assessed the use of a rapid and portable *S*. *haematobium* recombinase polymerase amplification (Sh-RPA) molecular diagnostic assay, coupled with simplified sample preparation methods that can be carried out in resourse-poor settings, to diagnose FGS using CVL and VSS samples. Performance of the Sh-RPA was excellent when compared to real-time PCR across all comparisons, even when using simplified sample preparation methods. Of note, optimal performance of the Sh-RPA may be achieved when used with the lesser-invasive VSS samples. The Sh-RPA therefore shows promise as a rapid and sensitive diagnostic tool capable of diagnosing FGS at the point-of-care in schistosomiasis-endemic settings.

## Introduction

Schistosomiasis is a neglected tropical disease (NTD) caused by infection with parasitic blood fluke trematodes of the genus *Schistosoma* that can lead to debilitating morbidity and mortality [1]. Whilst it is estimated that over 230 million people are currently infected globally, approximately 95% of all cases occur within sub-Saharan Africa [2]. Of these, around two-thirds are caused by *Schistosoma haematobium*, the causative parasite of urogenital schistosomiasis [3,4].

Pathologies associated with urogenital schistosomiasis occur primarily as a result of the copious number of eggs produced by female adult worms which inhabit the venous plexus of the bladder [5]. To perpetuate the parasite’s life cycle, these eggs penetrate blood vessel walls and migrate through surrounding tissues with the aim of reaching the bladder lumen for excretion and onward transmission. A large proportion of eggs however, fail to reach the bladder lumen and instead become sequestered throughout the urinary and reproductive organ tissues, evoking a T helper type-2 (Th2) cell-driven granulomatous response that often results in chronic inflammation, severe abdominal pain, tissue fibrosis and subsequent permanent organ damage which can potentially progress to bladder cancer [6–9].

In women, eggs deposited throughout the genital organs can cause genital itching, intravaginal lesions, vaginal discharge, dysuria, postcoital bleeding and destruction of the cervicovaginal mucosa [10–12]. In chronic cases, ectopic pregnancy, miscarriage and infertility can also occur [13–15]. These clinical manifestations of infection with *S*. *haematobium* are collectively termed female genital schistosomiasis (FGS) [13,16]. Further to the disabling pathology associated with FGS, *S*. *haematobium* infection has also been associated with sexually transmitted infections (STIs) [17,18], cervical dysplasia [19] and HIV transmission [20–25].

The World Health Organization (WHO) estimates that approximately 56 million women currently suffer from some form of FGS, though this is widely considered a gross underestimate owing to difficulties in infection and genital disease diagnosis [26–35]. Given these challenges, direct molecular diagnosis of FGS using the polymerase chain reaction (PCR) to detect *S*. *haematobium* ova-derived DNA within cervicovaginal lavage (CVL) samples has been evaluated, however scalability and feasibility of PCR using CVL samples is hindered by the complexitiy and level of expertise required for sample collection and processing [26,36–39]. The Bilharzia and HIV (BILHIV) study validated the novel use of genital self-sampling for real-time PCR analysis as a feasible approach to community-based diagnosis of FGS [40,41]. Here, it was reported that real-time PCR using lesser invasive genital self-swab sampling may provide a diagnostic accuracy of equal, or even improved, sensitivity when compared to real-time PCR using relatively invasive CVL sampling [40]. In addition, genital self-sampling can be performed by the patient within the home, whereas CVL sampling requires more invasive speculum insertion and must be carried out within a clinical setting by trained health workers.

Though highly sensitive and specific, PCR-based diagnostics are currently unsuited for use in most schistosomiasis-endemic settings [42]. Both PCR itself, as well as the essential preliminary steps needed to isolate DNA from clinical samples require expensive and sophisticated equipment, specialised personnel and reliable laboratory infrastructure seldom available in endemic areas. In addition, the provision of rapid test results, which cannot be delivered through use of PCR, is vital to support direct follow-up and targeted treatment, supporting compliance and effective interventions at the point-of-care.

Isothermal (single and constant temperature) DNA amplification methods offer an alternative to PCR-based amplification and are better suited for use in resource-poor settings as they require only minimal equipment that can be highly portable and user-friendly, can be performed using a simplified, or ‘crude’, DNA extraction processes easily prepared under field conditions, and can provide rapid and more easily-intpreted results (<1 hour from sample preparation to result) [42–44]. For these reasons, a variety of isothermal and field-deployable DNA amplification technologies have been developed and utilised for diagnostic purposes; the most common being Recombinase Polymerase Amplification (RPA) and loop-mediated isothermal amplification (LAMP). As an example, a previously developed RPA assay (the RT-ShDra1-RPA) has been used to detect trace levels of *Schistosoma* ova-derived DNA within egg-spiked laboratory samples (able to detect a single *S*. *haematobium* egg within 100 μl ddH_2_O), as well as in clinical urine samples from patients with very low infection intensities (1–3 eggs per/ 10 ml) [45–49].

Here, we aimed to assess the use of the RT-ShDra1-RPA assay (termed herein as Sh-RPA) for the diagnosis of FGS using CVL and vaginal self-swab (VSS) samples obtained during the BILHIV study, comparing Sh-RPA performance to that of real-time PCR as described previously [40]. In addition, various CVL sample preparation methods were evaluated with the aim of providing a straightforward and crude DNA extraction method to facilitate point-of-care testing in resource-poor schistosomiasis-endemic settings.

## Methods

### Ethics statement

Ethical considerations with regards to the initial study have been outlined previously [40]. All study participants gave written informed consent to participate in the initial study. All laboratory analysis performed here was carried out blinded from any other data. This study was approved by the University of Zambia Biomedical Research Ethics Committee (Reference: 011-08-17), the Zambia National Health Research Authority, and the London School of Hygiene and Tropical Medicine (LSHTM) Ethics Committee (References: 14506 & 17143).

### Study site and participants

All VSS and CVL samples analysed within this study were obtained as part of the BILHIV study based in central Livingstone, Southern province of Zambia, as described previously [40,41]. Between January and August 2018, non-pregnant, sexually active women between the ages of 18 and 31 years and resident in one of the two assessed communities were eligible and invited to participate in a cross-sectional FGS survey. In total, 603 eligible women provided both a VSS and cervical self-swab sample. Of these, 527 provided a CVL sample [40]. Due to financial and logistical constraints, only one form of genital self-swab sample, the VSS, was analysed by Sh-RPA within this study.

### Home-based vaginal self-swab (VSS) sampling, clinic-based cervicovaginal lavage (CVL) sampling and DNA extraction/real-time PCR analysis

During home visits, trained community health workers provided instructions for VSS sampling as described previously [40]. Study participants who were not currently menstruating were also invited to attend the Livingstone Central Hospital cervical cancer screening clinic where one of two trained midwives performed a CVL [40]. Collected VSS specimens were placed in a cool box for transportation to the Zambart laboratory and immediately stored at -80°C upon arrival. CVL samples were placed immediately on ice after collection and were also then transported to the Zambart laboratory for storage at -80°C.

VSS and CVL samples were then transported to the Leiden University Medical Center (LUMC) under frozen conditions for DNA extraction. Samples were processed using *QIAamp Mini kit* spin columns (QIAGEN Benelux, Venlo, The Netherlands) as described previously [40], providing ‘pure’ (Pu) DNA preparations using VSS (n = 603) and CVL (n = 527) samples termed here as ‘PuVSS’ and ‘PuCVL’ preparations, respectively. These preparations were initially used for real-time PCR analysis as reported in [40] and were then transported to the Natural History Museum (NHM), London, UK under ambient conditions for Sh-RPA analysis. Real-time PCR data obtained during this initial study was used to determine which VSS and CVL samples would be used for Sh-RPA analysis.

### CVL and VSS sample inclusion criteria for Sh-RPA analysis

All fourteen real-time PCR FGS-positive CVL samples (2.7% of 527 total samples: C_t_ mean: 32.8; C_t_ range: 23.6–38; C_t_ median: 34.55 (513 samples negative by real-time PCR)) and all 15 real-time PCR FGS-positive VSS samples (2.5% of 603 total samples: C_t_ mean: 31.9; C_t_ range: 23.1–39.4; C_t_ median: 34.3 (588 samples negative by real-time PCR)) [40] were purposefully selected for Sh-RPA analysis together with at least 20% randomly selected samples that were FGS-negative by real-time PCR when using both CVL and VSS samples. The number of samples used for each assessment varied depending on available DNA extraction and Sh-RPA resources. Only sample IDs were used during processing. As such, all samples were analysed blind to any associated data.

### ‘Crude’ DNA extraction from CVL samples

After the initial ‘pure’ DNA extraction procedure, approximately 800 μl of the original CVL sample material was available for further DNA extraction and analysis. DNA was extracted from the remaining material using two commercially available ‘crude’ DNA extraction methods: 1) the *SpeedXtract Nucleic Acid Kit* (QIAGEN, Germany) and 2) the *Extracta DNA Tissue Prep Kit* (Quantabio, USA). These samples are herein referred to as ‘crude’ (Cr) DNA preperations. These crude extraction methods were used as both provide a rapid extraction process that can be easily carried out in resource-poor field settings, essential for point-of-care molecular testing in many schistosomiasis-endemic areas. All crude DNA preparations were carried out at the LUMC with two negative extraction control samples (ddH_2_O) randomly incorporated into each extraction batch of 48 samples. No further material was available from VSS samples for any comparative extraction testing.

#### SpeedXtract DNA isolation procedure used to obtain crude SpeedXtract CVL (CrSpCVL) samples

To isolate DNA from CVL samples using the *SpeedXtract* Nucleic Acid Kit (QIAGEN, Germany), (a DNA isolation method specifically designed for use with isothermal assays and so not considered suitable for use with real-time PCR), a modified extraction procedure was carried out based on that used for urine samples as outlined previously [47,48]. Prior to extraction, CVL samples were thawed at room temperature and inverted to resuspend the sample. 50 μl of each CVL sample was then added to 100 μl EN buffer followed by 7.5 μl magnetic bead suspension solution A. Each extraction mix was vortexed for 15 seconds and incubated at room temperature for three minutes. All Eppendorf tubes containing the extraction mix were then transferred to a magnetic separation rack and left for one minute to allow magnetic bead (bound with DNA) aggregation. After one minute, the supernatant was gently removed using a micropipette, taking care not to disturb the aggregated magnetic bead pellet. 100 μl of SL buffer was then added and the extraction mix was again vortexed for 15 seconds, incubated at 95°C for 5 mins and then transferred back to the magnetic separation rack for one minute. The supernatant, now containing isolated DNA, was again removed using a micropipette and aliquoted into a new labelled eppendorf for storage and Sh-RPA testing. In total, 223 CrSpCVL samples (42.3% of all 527 CVL samples) were prepared using the *SpeedXtract* extraction method; 14 of which were purposely selected as these were positive for FGS by real-time PCR and 122 of which were randomly selected from the 513 CVL samples negative for FGS by real-time PCR [40]. Because additional reagents were available, a further 87 CrSpCVL samples were also prepared using CVL samples randomly selected from the remainding 391 CVL samples negative for FGS by real-time PCR.

#### Extracta DNA isolation procedure used to obtain crude Extracta CVL (CrExCVL) samples

To isolate DNA from CVL samples using the *Extracta* DNA Tissue Prep Kit (Quantabio, USA), 20 μl of each thawed CVL sample was added to 100 μl Extraction Buffer. The extraction mix was vortexed for 15 seconds, heated for 10 minutes at 95°C to lyse cells, and then allowed to cool to room temperature. 100 μl Stabilisation Buffer was then added to prevent DNA degradation. A total of 136 CrExCVL samples were prepared using the *Extracta* extraction method. Fourteen of these were purposely selected as these were positive for FGS by real-time PCR and the same 122 CVL samples randomly selected previously (during 2.4.1) also underwent DNA extraction using the *Extracta* method.

### Recombinase Polymerase Amplification (RPA) analysis

RPA analysis was carried out using a previously developed assay that has been used to detect *S*. *haematobium* DNA within urine samples (the RT-ShDra1-RPA assay) [47,48], here referred to as the ‘Sh-RPA’. The Sh-RPA was used to assess all CVL and VSS sample preparations derived from each extraction method; 1) PuCVL, 2) PuVSS, 3) CrSpCVL and 4) CrExCVL. Sh-RPA reactions were performed using TwistDx (Cambridge, UK), RPA Exo Kits. Each reaction (50 μl total) contained 2.1 μl of each primer (forward and reverse; 10 pmol), 0.6 μl internal probe (10 pmol), 29.5 μl rehydration buffer, 2.5 μl (280 nM) magnesium acetate (MgAc), 0.5 μL betaine (5 M betaine (720 mM) (B0300; Sigma-Aldrich, Gillingham, UK), 5 μl of extracted DNA, 7.7 μl of ddH_2_O and the TwistDX lyophilised RPA pellet.

Reactions were carried out by preparing a reagent master mix containing forward and reverse primers, internal probe, rehydration buffer, betaine and ddH_2_O. A total of 42.5 μL master mix was added to each lyophilised RPA pellet, followed by 5 μL of the DNA extract. Next, 2.5 μL MgAc was pipetted into each reaction tube lid, which was then carefully closed so that the MgAc, which initiates the RPA reaction, was not introduced into the reaction mix prematurely. Reaction tubes were inverted by hand to mix reagents and to initiate the RPA reaction. Tubes were then promptly centrifuged and placed into position within the AmpliFire isothermal fluorometer (Douglas Scientific, USA) [45], which was pre-heated to 42°C. All reactions were then run for 20 minutes according to pre-programmed assay conditions [45]. After 4 minutes, reaction tubes were temporarily removed from the AmpliFire testing device, inverted by hand to resuspend reagents, centrifuged and promptly returned to the device to continue amplification.

The AmpliFire testing device is capable of processing up to eight samples per assay run (20 min), and so a fresh master mix was prepared every other assay run (i.e., each master mix was sufficient for 16 samples). Reactions were prepared and run in groups of 16 samples (2 assay runs), which consisted of 13 test samples (5 μL DNA), one positive control (1 μL DNA extracted from a single *S*. *haematobium* egg using the *SpeedXtract* protocol + 9 μL ddH_2_O) in a known and standard position, one negative control (10 μL no template ddH_2_O [45]) in a known and standard position and one additional negative control (10 μL no template ddH_2_O) placed in a known but random position amongst the 13 test samples.

**Fig 1:**
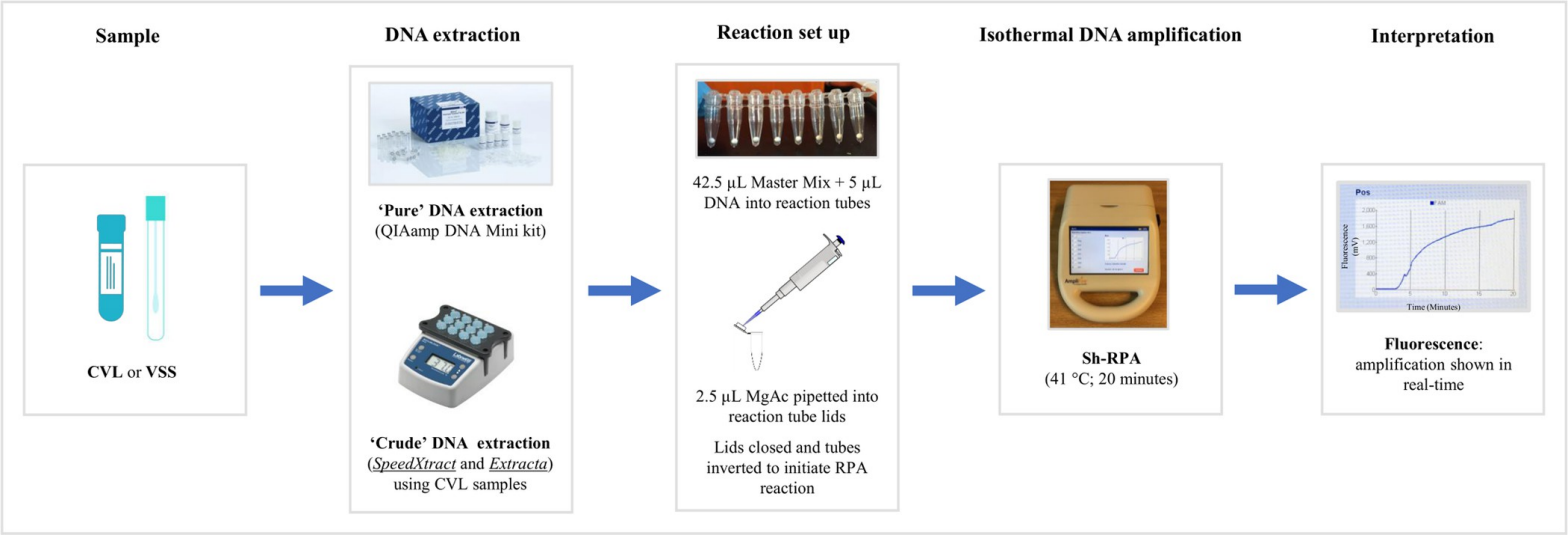
Schematic showing steps involved in Sh-RPA analysis.

Samples were considered positive if florescence increased beyond a threshold marker (>200 mV increase form baseline) estimated based on any background fluorescence detected using negative controls [48]. Samples were considered negative if amplification curves did not cross this threshold marker. A schematic outlining the primary steps involved in Sh-RPA analysis can be seen in Fig 1.

### Total number of samples analysed using Sh-RPA

A total of 190 PuVSS samples and 133 PuCVL samples were analysed using Sh-RPA at the NHM. Of the 223 CrSpCVL samples, 142 were analysed using Sh-RPA at the LUMC. All 223 CrSpCVL samples were then transported to the NHM at ambient temperature, where the remaining 81 CrSpCVL samples were analysed by Sh-RPA. For quality control purposes, at the NHM, Sh-RPA analysis was repeated using a random subset (n = 55) of CrSpCVL samples that had already been analysed by Sh-RPA at the LUMC. All 136 CrExCVL samples were transported to the NHM at ambient temperature and analysed by Sh-RPA.

### Data analysis

To assess the diagnostic performance of the Sh-RPA assay compared to real-time PCR (considered here as the reference assay), sensitivity and specificity values were calculated using the *epiR* package [50] within R version 1.3.959 [51]. Predictive values are not reported here as these are not suitable when using known and preselected real-time PCR positive and negative samples.

## Results

**Fig 2:**
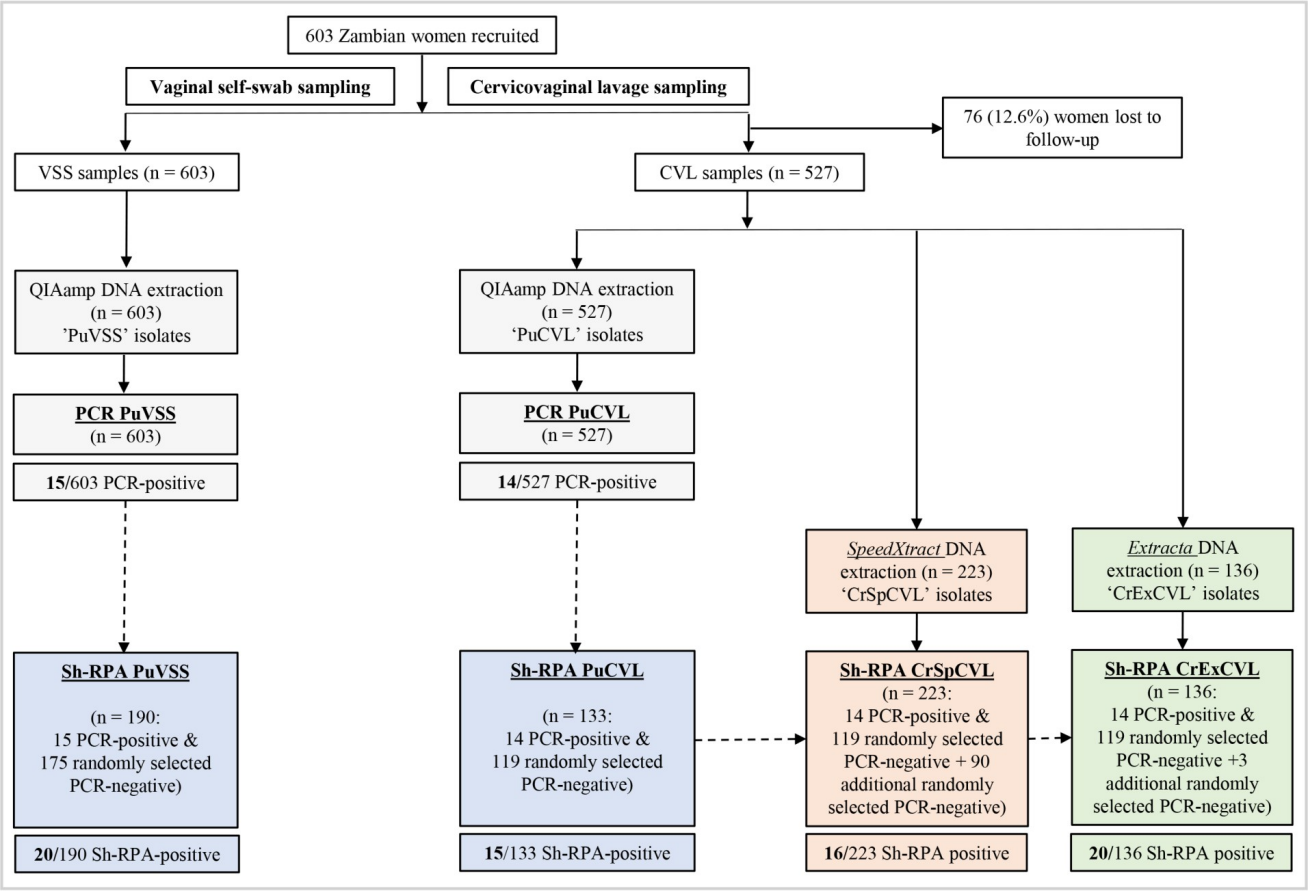
Flow schematic outlining participant recruitment, clinical samples provided by patients, DNA isolation methods used and molecular diagnostic assessment (real-time PCR and Sh-RPA). DNA isolation and real-time PCR carried out by Sturt *et al*., [40] is coloured grey. DNA isolation and Sh-RPA carried out here is coloured according to DNA extraction method used: *QIAamp Mini Kit*: blue, *SpeedXtract*: orange, *Extracta*: green. Dashed arrows are used to illustrate that real-time PCR data obtained during [40] was used to determine which VSS and CVL samples would be selected for Sh-RPA analysis. Sh-RPA performance and sample preparation comparisons are detailed in Table 1.

A flow schematic outlining participant recruitment, clinical samples provided by patients, DNA isolation methods used and molecular diagnostic assessment (real-time PCR and Sh-RPA) can be seen in Fig 2.

Of note, sensitivity of the Sh-RPA assay ranged between 71.4 (95% CI: 41.9–91.6) and 85.7 (95% CI: 57.1–98.2) across all extraction methods used to isolate DNA from CVL samples when compared to real-time PCR using PuCVL samples. In particular, Sh-RPA using CrSpCVL samples had a sensitivity of 85.7% (95% CI: 57.1–98.2) when compared to real-time PCR using PuCVL samples. The results also suggest that optimal performance may be achieved when the Sh-RPA is used with PuVSS samples; yielding a sensitivity of 93.3% (95% CI: 68.1–100) when compared to real-time PCR using the same PuVSS samples. Specificity of the Sh-RPA was >91% across all comparisons.

**Fig 3:**
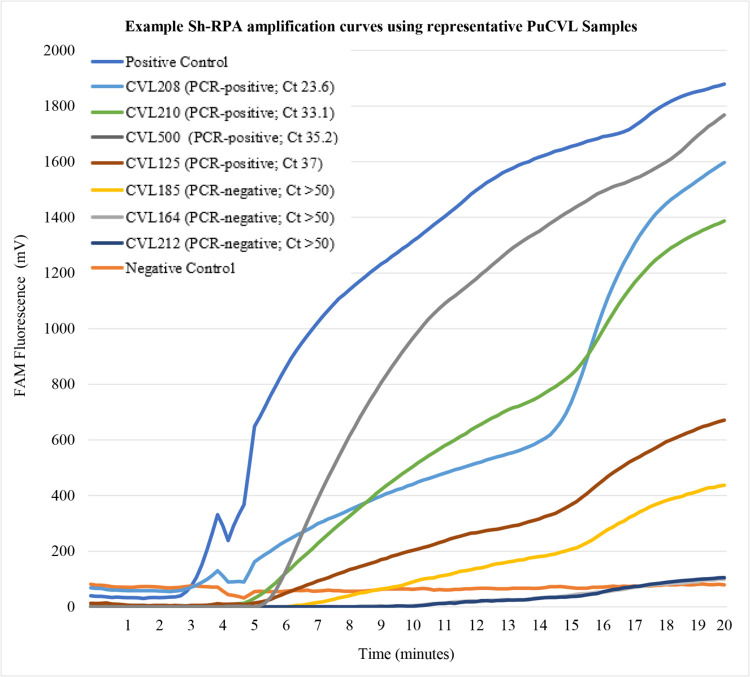
Example Sh-RPA amplification curves using representative PuCVL samples. Three strong and early-onset positive Sh-RPA amplification curves with relatively low associated real-time PCR C_t_ values are shown (samples CVL208, CVL210 and CVL500). One weak but positive Sh-RPA amplification curve with a relatively high associated real-time PCR C_t_ value is shown (CVL125). One weak but positive Sh-RPA amplification curve with an associated real-time PCR C_t_ value of >50 (and so deemed negative by real-time PCR [40]) is shown (CVL185). Samples CVL164 and CVL212 were deemed negative by both real-time PCR (C_t_ >50) and Sh-RPA. Neither sample generated a Sh-RPA amplification curve. All real-time PCR C_t_ values were generated during [40].

Although the Sh-RPA is not considered a fully quantitative diagnostic methodology, it is worth noting here that upon visual analysis, samples with lower real-time PCR C_t_ values (and so higher concentrations of DNA) appeared to give strong and well-defined Sh-RPA amplification curves. In addition, the onset of Sh-RPA amplification curves was earlier in samples containing higher concentrations of DNA (as verified using real-time PCR C_t_ values). Examples of typical Sh-RPA amplification curves with respect to real-time PCR C_t_ values can be seen in Fig 3. When analysing samples deemed positive by real-time PCR but negative by Sh-RPA, there did not appear to be any association between high real-time PCR C_t_ values (and so low concentrations of DNA) and false Sh-RPA-negatives.

## Discussion

Reliable, rapid and non-invasive methods of diagnosing FGS at the point-of-care are sorely needed to promptly identify women suffering from, or at risk of, any FGS-related pathologies. Though direct diagnosis using PCR can be sensitive, even when using self-administered vaginal and cervical self-swab samples, PCR is currently unsuited for use at the point-of-care. Here, we assessed the use of a previously developed rapid and portable RPA assay (the RT-ShDra1-RPA, termed here as ‘Sh-RPA’) for the diagnosis of FGS in Zambian women using cervicovaginal lavage samples and lesser invasive vaginal self-swab samples. In addition, various CVL sample preparation methods were evaluated for use with the Sh-RPA assay.

As the same PuCVL and PuVSS DNA sample isolates were used during real-time PCR and Sh-RPA analysis, a direct comparison can be made between both diagnostic methods. When comparing Sh-RPA to real-time PCR using PuCVL samples, 10 of 14 real-time PCR-positive samples were also positive by Sh-RPA. As such, sensitivity was 71.4%, suggesting further optimisation of the Sh-RPA may be needed as four of 14 samples positive by real-time PCR were missed by Sh-RPA. When vaginal swabs were used with the same extraction method (PuVSS), sensitivity was improved (93.3%) as 14 of 15 real-time PCR-positive samples were positive by Sh-RPA. This may be because genital swabs come into direct contact with *S*. *haematobium* DNA (either as sequestered eggs or cell-free DNA) and because genital swabs are abrasive when collecting cells from tissue surfaces, which is not the case for CVL sampling. It may also be because DNA collected using genital swab samples will likely be more concentrated when compared to DNA collected using CVL samples, which uses a solution of 10 ml saline solution flushed across the cervix and vaginal walls [41], or because DNA collected using genital swabs is better preserved prior to DNA isolation than when collected using CVL. This discrepancy, however, requires formal quantification to clarify and this is warranted as genital self-swab sampling is far more field-appropriate, acceptable to patients, and scalable when compared to CVL sampling and does not appear to be any less sensitive [40]. Specificity of the Sh-RPA compared to real-time PCR was high when using both PuCVL and PuVSS samples (95.8% and 96.6%, respectively). It should be noted, however, that any samples positive by Sh-RPA but negative by real-time PCR may potentially be positive cases missed by real-time PCR.

Interestingly, when used with PuVSS samples, the Sh-RPA had a higher sensitivity when compared to real-time PCR using PuCVL samples (64.1%) than real-time PCR had using the same PuVSS samples (35.7%) [40]; further highlighting that genital self-swabs can be used to successfully diagnose FGS as demonstrated previously [40]. Specificity, however, was reduced, though this is because four samples deemed negative by real-time PCR using PuVSS samples were deemed positive by Sh-RPA using the same PuVSS samples. Although further optimisation of the Sh-RPA is needed, these results supplement previous findings and too demonstrate the potential of the Sh-RPA assay as a reliable means of performing rapid molecular diagnosis at the point-of-care in resource-poor settings [48].

Without a rapid, cost-effective and field-approptiate DNA extraction method that provides a suitable DNA preparation, molecular assays cannot be deployed at the point-of-care in resource-limited settings. DNA extraction techniques must be straightforward to carry out, affordable, and meet high standards with regards to quality, purity and concentration. Thus, diagnostic sample choice and sample preparation are major components of any field-based molecular diagnostic procedure. These aspects, however, can be overlooked, with the primary focus of studies often being on only the molecular assay’s performance.

Here, it was found that the total time needed to carry out the *QIAamp Mini kit* DNA extraction method using 20 samples, from sample preparation to completion, is approximately two hours and the total cost is approximately $6 USD per sample. The total time needed to carry out the *SpeedXtract* extraction method using 20 samples, from sample preparation to completion, is approximately 15 minutes and the total cost is approximately $1.5 USD per sample. The total time needed to carry out the *Extracta* extraction method using 20 samples, from sample preparation to completion, is approximately 5–10 minutes and the total cost is approximately $0.5 USD per sample. In addition, once DNA has been isolated, we estimate that the Sh-RPA costs approximately $4 USD per sample and between $5,000 and $10,000 USD for a portable and isothermal RPA device (depending on the device), whilst real-time PCR costs approximately $1.5 USD per sample and between $10,000 and $30,000 USD for a real-time PCR thermocycler (depending on the thermocycling equipment and any additional equipment such as a personal computer). Whilst these are informal speed and cost estimates (that include all reagents, plasticwear and equipment required for DNA extraction, real-time PCR and Sh-RPA), a more comprehensive investigation into optimal DNA extraction methods for use with the Sh-RPA, and how these compare to real-time PCR in terms of speed, ease-of-use, total cost, portability and quality, is required. In addition, it is worth highlighting here that as these methods continue to be developed and revised, reagent and equipment costs are likely to be reduced over time.

With regards to diagnostic performance, of the three DNA extraction methods used to isolate DNA from CVL samples, the *SpeedXtract* method had the greatest sensitivity and specificity when comparing the Sh-RPA to real-time PCR using PuCVL samples (Table 1). This is a highly relevant finding as the *SpeedXtract* extraction method is a rapid DNA extraction process that can be easily carried out in resource-poor field settings, unlike the *QIAamp Mini kit*, which requires sophisticated and robust laboratory infrastructure [42,48]. In addition, the *Extracta* DNA extraction method, which can also be easily and rapidly carried out in resource-poor field settings, used with Sh-RPA had an identical sensitivity to PuCVL samples used with Sh-RPA, when both were compared to real-time PCR using PuCVL samples. This too is a highly relevant finding, illustrating that both of these rapid, low-resource and field-applicatble DNA extraction methods can be used to reliably extract DNA from CVL samples. It should also be noted here that whilst the *SpeedXtract* method is a bead-based procedure that concentrates cells and DNA, the *Extracta* method is a relatively simple lysis procedure that does not concentrate DNA. As such, the *SpeedXtract* method may perform better than the *Extracta* method when used with high volume samples such as CVL. This, however, also requires additional analyses to clarify. Nevertheless, these results demonstrate the reliability of both *SpeedXtract* and *Extracta* crude DNA extraction methods; both of which therefore show promise for future use as a means of carrying out simplified and rapid molecular FGS diagnosis within any health facility capable of collecting CVL samples [48].

**Table 1:** Sh-RPA performance and sample preparation comparisons using DNA isolated from CVL and VSS samples by *QIAamp Mini Kit* (coloured blue), *SpeedXtract* (coloured orange) and *Extracta* (coloured green), extraction methods.

Reference standard (Sample preparation)	Index test (Sample preparation)	Sensitivity % [TPI /PR]** (95% CI)	Specificity % [TNI /NR]** (95% CI)
Real-time PCR* (PuCVL)	**Sh-RPA** (PuCVL)	**71.4** [10/14] (41.9–91.6)	**95.8** [114/119] (90.5–98.6)
	**Sh-RPA** (CrSpCVL)	**85.7** [12/14] (57.1–98.2)	**98.1** [205/209] (95.1–99.5)
	**Sh-RPA** (CrExCVL)	**71.4** [10/14] (41.9–91.6)	**91.8** [112/122] (85.4–96)
	**Sh-RPA** (PuVSS)	**64.1** [9/14] (35.1–87.2)	**93.7** [164/175] (89.7–97.2)
Real-time PCR* (PuVSS)	**Sh-RPA** (PuVSS)	**93.3** [14/15] (68.1–100)	**96.6** [169/175] (92.7–98.7)
**Sh-RPA** (PuCVL)	**Sh-RPA** (PuVSS)	**71.4** [10/14] (41.9–91.6)	**93.1** [108/116] (86.9–97)

*Analysis carried out in [40].

**Where: ‘TPI’ denotes true positive index test results, ‘TNI’ denotes true negative index test results, ‘PR’ denotes positive reference test results and ‘NR’ denotes negative reference test results.

### Study limitations and future work

The primary limitation of this study is the low proportion of FGS-possitive CVL and VSS samples by real-time PCR when compared to FGS-negative CVL and VSS samples by real-time PCR, and the limited number of samples assessed using Sh-RPA. As a result of this, very wide 95% confidence intervals are given for all sensitivity values. Future studies should therefore use a greater number of CVL and VSS samples that are FGS-positive by real-time PCR and also aim to assess a greater number of samples by Sh-RPA in order to achieve the precision needed to draw clear conclusions about Sh-RPA performance. For example, by doing so, more precise conclusions regarding the optimal clinical sample to use with the Sh-RPA (CVL vs VSS), the optimal DNA isolation method(s) to use, and any associations between real-time PCR C_t_ values and Sh-RPA amplification curve profiles can be drawn.

Another important limitation of this study is that only one DNA extraction method could be used to isolate DNA from VSS samples, limiting our conclusion about the valitidity of different extraction methods. Given the encouraging performance of the *SpeedXtract* and *Extracta* DNA extraction methods when used with CVL samples, future work should assess the diagnostic performance of the Sh-RPA assay when used with DNA isolated from the lesser-invasive VSS (or cervical self-swab, [40]) samples using these extraction methods. In addition, as *SpeedXtract* and *Extracta* DNA extraction methods have not yet been validated for use with real-time PCR, a direct comparison between both diagnostic assays using these samples could not be performed.

For quality control and quality assurance, future work should also be carried out to incorporate internal Sh-RPA assay inhibition controls to ensure that all Sh-RPA positive outcomes are not a result of assay or sample preparation contamination or faults [48]. This could either be an internal DNA extraction control like that used for real-time PCR [40] or a human DNA target that can be amplified in tandem with *S*. *haematobium* DNA within a multiplex assay. Furthermore, *S*. *haematobium* DNA-negative samples, provided by uninfected individuals, should also be incorporated into future assays to ensure no chance of non-target DNA amplification and to aid in result interpretation with respect to the Sh-RPA infection threshold marker. Incorperating DNA-negative donor samples will also support the development of flourscent threshold algorithms that can be used to automatically assign samples as positive or negative. Algorithms such as these are routinely programmable into flurometers used for RPA such as the Amplifire testing device used here.

Whilst specificity values of > 91% were given across all comparisons, Sh-RPA sensitivity values ranged between 64.1% and 93.3% (Table 1). These data suggest that additional tailoring and refinement of the Sh-RPA assay should be carried out to further improve diagnostic performance of the assay when used to diagnose FGS. Once improved, future assessment of the Sh-RPA assay should be performed in schistosomiasis-endemic settings at the peripheral level (e.g., directly in health facilities or mobile laboratories) to fully evaluate its potential as a portable and robust diagnostic assay, suitable for use at the point-of-care [44]. Furthermore, diagnostic performance of the Sh-RPA should also be compared to that of alternative isothermal point-of-care molecular diagnostic assays, such as the LAMP assay [45], to support the future development and advancement of point-of-care molecular diagnostics as well as other diagnostics used to detect active infection. Examples of these include urine-egg microscopy [48] and the highly-sensitive up-converting particle-lateral flow test for the detection of circulating anodic antigen (UCP-LF-CAA) [52,53].

As found previously, it was also suggested here that the time of onset of Sh-RPA amplification curves is earlier in samples containing greater concentrations of DNA (as verified using real-time PCR C_t_ values) [48]. Future work to clarify any association between DNA concentration and the onset of Sh-RPA amplification, as well as between DNA concentration and the quality of Sh-RPA amplification curves, is therefore warranted.

## Conclusions

Here, we demonstrate that the rapid and portable Sh-RPA assay is able to reliably detect and amplify *S*. *haematobium* DNA within cervicovaginal lavage and vaginal self-swab samples. Additionally, and importantly, the Sh-RPA assay was also able to reliably detect *S*. *haematobium* DNA within DNA isolated from CVL samples using two crude extraction methods that can be easily and rapidly carried out in resource-poor schistosomiasis-endemic settings. These results supplement previous findings, highlighting that the use of genital self-swab sampling for diagnosing FGS should be explored further [40] whilst also demonstrating that rapid and portable DNA isolation methods can be used to detect *S*. *haematobium* DNA within clinical samples using RPA [49].

Although additional development and assessment is needed before the upscaled and routine diagnostic use of the Sh-RPA assay, the assay proved highly portable and would perform well as a part of a mobile laboratory in resource-poor settings. As such, with further development, the Sh-RPA shows excellent promise for future use as a means of reliably diagnosing FGS at the point-of-care and so could not only contribute to a better understanding of FGS prevalence across sub-Saharan Africa, but could also aid in reducing the degree of debilitating disease-related morbidities experienced by those in endemic areas.
